# Advances in Food Processing Techniques for Allergenicity Reduction and Allergen Identification

**DOI:** 10.3390/foods14223933

**Published:** 2025-11-17

**Authors:** Marta Wójcik, Krystian Marszałek, Edyta Juszczuk-Kubiak

**Affiliations:** 1 Department of Fruit and Vegetable Product Technology, Wacław Dąbrowski Institute of Agricultural and Food Biotechnology—State Research Institute, 02-532 Warsaw, Poland; marta.wojcik@ibprs.pl; 2 Department of Biotechnology, Wacław Dąbrowski Institute of Agricultural and Food Biotechnology—State Research Institute, 02-532 Warsaw, Poland

**Keywords:** food allergy, allergenic proteins, food processing, allergen detection, food safety

## Abstract

Food allergies represent a growing global public health concern, affecting individuals of all ages in developed and developing countries. Complete avoidance of allergenic foods remains challenging, underscoring the need for effective strategies to reduce food allergenicity. Consequently, there is increasing interest in techniques that lower the immunoreactivity of allergenic proteins in food. The use of processes such as thermal treatment, enzymatic treatment, or fermentation can lead to structural changes in proteins, limiting their ability to bind IgE antibodies. However, the effectiveness of these methods varies and depends on both the type of protein and the conditions of the process used. At the same time, it is crucial to select the appropriate method for determining protein immunoreactivity, as there is no single, universal measurement tool. Differences in sensitivity and detection range between methods, such as ELISA, Western blot, and LC-MS/MS, may affect the reproducibility of the results obtained. The development of a “hypoallergenic” product remains a major challenge, highlighting the need for an integrated approach combining a variety of technological strategies. The aim of this article is to review the available food processing methods that reduce protein allergenicity and to analyze the appropriate selection of analytical tools for their evaluation.

## 1. Introduction

Food allergy (FA) is an atypical immune reaction triggered by ingesting specific food products containing allergenic proteins [1,2]. In recent years, there has been a significant rise in the prevalence of allergic diseases, including food allergies [3,4]. While these conditions can impact individuals across all age groups, they are most frequently observed in young children, primarily due to their immature digestive systems [5,6,7]. A significant factor contributing to the increasing incidence of allergies is the consumption of highly processed foods containing preservatives, food dyes, and other chemical additives. Moreover, the disruption of the gut microbiome’s diversity and metabolic activity, resulting from excessive hygiene, frequent antibiotic use, or insufficient dietary fiber, may substantially aggravate allergic symptoms [5,6,7,8,9,10,11,12].

In 2014, Regulation (EU) No 1169/2011 of the European Parliament and of the Council on the provision of food information to consumers expanded the list of recognized allergens to include 14 items: cereals containing gluten, crustaceans, eggs, peanuts, tree nuts, fish, soy, mustard, sesame, milk, celery, lupin, and sulfur dioxide [5,13,14]. The reported prevalence of food allergies varies by geographical region and depends on data collection and analysis criteria [15,16,17]. However, it is unclear how significant these differences are, as studies in different countries often use various methodologies, sampling techniques, and food products evaluated [18,19,20,21]. Nonetheless, the notable rise in food allergies presents a significant challenge to both society and clinical practice. The prevalence of food allergies also varies considerably across continents and even between individual countries. For example, data published by the National Health Interview Survey (NHIS) indicate that clinically diagnosed food allergies affect 6.2% of American adults and 4.5% of Asians [22]. A medical report from the Canadian Primary Care Sentinel Surveillance Network revealed that 2.53% of children in Canada have clinically diagnosed food allergies [23]. Conversely, Brazil’s food allergy data vary widely. Studies indicate that about 1% of the population has clinically diagnosed food allergies, while self-report surveys suggest that as many as 10%. Eight percent believe they have food allergies. Silva et al. [24] showed that food allergies impact 3% to 6% of children and 1% to 2% of adults. Lyons et al. [25] noted that the prevalence of food allergies, defined by symptoms and confirmed through positive diagnostic tests, ranges between 1.9% and 5–6% in children and adults. Additionally, research conducted by Barth et al. [26] from 2021 to 2023 reveals a significant gap between clinically confirmed food allergies and those reported by individuals themselves. In Poland, the number of clinically confirmed adult cases is 2.8% [27,28]. Among children, the age distribution is as follows: <12 months (3.63%), 1–5 years (39.54%), 6–13 years (46.32%), and 14–18 years (10.0%) [28].

The increasing prevalence of food allergies underscores the need to remove allergens from the human diet. Pharmacological treatment only alleviates the side effects of food allergies during immunotherapy. Consequently, developing innovative food processing techniques becomes essential. Recent evidence suggests that various food processing methods, such as ultrasound processing, irradiation, chemical modification, high-pressure treatment, cold plasma, or microbial fermentation, are promising approaches to reducing the immunoreactivity of allergen proteins in food products [29,30]. These techniques can alter protein structures and decrease allergenicity while maintaining the organoleptic properties and quality of the food products [30,31,32]. Current research on the immunoreactivity of food proteins, particularly regarding alternative protein sources, evaluates their ability to trigger an IgE-dependent response and examines the structural and functional changes induced by processing technologies. Allergen detection relies on immunological, spectroscopic, and bioinformatic methods, continuously improving the accuracy of predicting allergic risks and devising strategies to mitigate them. The primary methods for detecting IgE include tests such as ELISA and Western blot. These methods enable the assessment of specific IgE antibodies, allowing for the precise identification of allergenic epitopes in individuals with allergies [30,31,32,33,34,35,36,37]. They also provide insights into allergic reactions under physiological conditions. Modern instrumental techniques, including Fourier transform infrared spectroscopy (FTIR) and Liquid chromatography tandem mass spectrometry (LC-MS/MS), are employed depending on the structural alterations of allergens.

This review systematically evaluates recent advances in food processing techniques aimed at reducing the allergenic potential of food products and discusses analytical methods for detecting allergenic proteins. By addressing both technological and analytical perspectives, this work provides a comprehensive overview of how processing-induced structural modifications influence protein immunoreactivity and how these changes can be effectively monitored. Particular emphasis is placed on innovative, integrated approaches that combine processing and detection methods, enabling the tracking of changes in protein immunoreactivity. It is worth emphasizing that a comprehensive approach to these issues is essential for reliable food safety assessment. Therefore, this review highlights the need for an integrated approach that directly links technological processes affecting protein structure with their quantitative and qualitative analytical assessment.

## 2. Origin of Food Allergens

**Figure 1:**
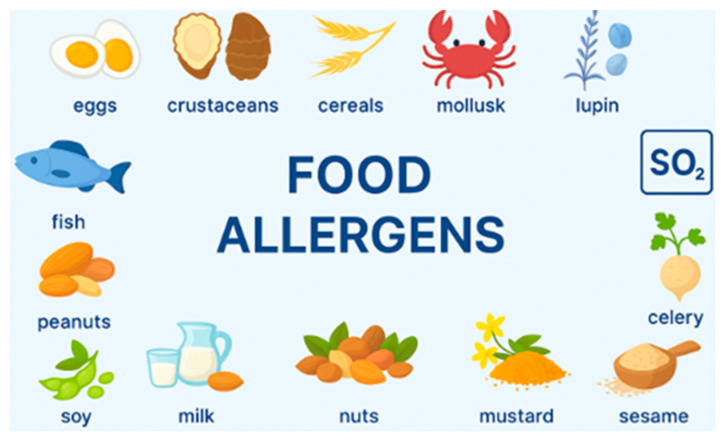
The fourteen most common food allergens are listed in Regulation (EU) No 1169/2011 [41]. Drawing by authors with Canva Pro software.

Food allergens are proteins that can trigger an immune response in individuals who are sensitive to them. The mechanisms of allergenicity often involve these proteins binding to immunoglobulin E (IgE) antibodies, causing the release of histamine and other inflammatory mediators [38,39,40,41,42]. Symptoms can range from mild reactions, such as skin hives and gastrointestinal discomfort, to severe anaphylaxis, which can be life-threatening [38,39,40]. Food allergens are categorized based on their origin, mainly into plant-based and animal-based allergens [Figure 1] [43]. Plant-based allergens occur in various foods, including fruits, vegetables, legumes, nuts, and grains. Peanuts and soybeans are two of the most significant sources of allergens. Peanuts contain several allergenic proteins, such as Ara h 1, Ara h 2, and Ara h 3 [44]. Likewise, soy allergens, especially Gly m 4 and Gly m 5, are common triggers for allergic reactions [45]. Tree nuts, such as walnuts, cashews, and almonds, are a potent and common source of food allergens that cause IgE-mediated allergic reactions. Each type of nut contains distinct proteins capable of provoking allergic responses. For example, peanuts contain an allergenic protein called Ara h 1 [46]. Certain fruits, such as bananas, kiwi, melons, and peaches, as well as vegetables like celery and carrots, contain allergenic proteins that can trigger oral allergy syndrome (OAS), particularly in individuals who are sensitive to pollen [47]. For example, proteins such as Pru p 3 from peaches can cross-react with pollen allergens [48]. Furthermore, gluten, a protein found in wheat, barley, and rye, can trigger allergic reactions in susceptible individuals [49].

Conversely, animal-based allergens primarily originate from meats, seafood, and dairy products, resulting in various allergic reactions. Milk allergy is often observed in infants and children and is associated mainly with proteins such as casein and whey. Among shellfish, crustaceans such as shrimp, crab, and lobster are primary causes of allergic reactions, primarily due to proteins like tropomyosin [50]. Fish allergies can be caused by various species, with certain fish (like mackerel and tuna) more likely to provoke reactions [51]. Allergies to meat (such as beef or pork), although less common than allergies to dairy or seafood, can develop and are often linked to specific proteins found in the meat (Bos d 6, myoglobin, actin, tropomyosin) [52,53,54]. Furthermore, allergy is mediated by cross-reactivity resulting from the similar molecular structures of aeroallergens and food-derived allergens [55]. Therefore, understanding the thresholds at which allergic reactions occur is critical for developing effective strategies to prevent and manage them. The findings of a study by Remington et al. [56] provide updated values for the minimum doses required to induce allergic reactions for fourteen priority food allergens [Table 1].

The extensive longitudinal study conducted from 2011 to 2018 with over 3400 participants aimed to determine the ED01 and ED05 values, representing the doses at which 1% and 5% of the allergic population would experience objective allergic reactions [56,57]. The allergens investigated included eggs, cow’s milk, wheat, hazelnuts, peanuts, soy, lupin, fish, and shellfish, providing a comprehensive understanding of the varying allergenic potentials of these foods. The results highlight significant variability in allergy thresholds across different food products. Notably, the study documented the lowest threshold doses, ranging from 0.1 to 0.5 mg of protein for highly allergenic foods such as eggs, cow’s milk, hazelnuts, and peanuts, underscoring the need for heightened awareness regarding even small amounts of these allergens [52,53,54,55,56,57]. In contrast, shellfish exhibited the highest threshold doses, ranging from 2.7 to 166 mg of protein, indicating that larger quantities are required to provoke allergic responses compared to other allergens. This introduction sets the stage for a detailed exploration of the implications of these findings for allergy management and risk assessment in the food industry.

A detailed data analysis reveals that completely eliminating the risk of allergic reactions remains extremely challenging. The ED01 threshold levels for walnuts (0.03 mg of protein) and cashews (0.05 mg of protein) are very low. This suggests that even small amounts of residual, partially broken-down proteins can still pose a significant risk to consumers. Such low ED01 levels challenge modern processing methods to create “hypoallergenic” products. Unfortunately, even advanced physicochemical techniques, such as protein denaturation, enzymatic hydrolysis, glycation, or oxidation, may not fully eliminate the immunoreactive potential of the proteins. An additional difficulty is that new epitopes may form during processing, which can still bind to IgE antibodies, further complicating safety assessments. Therefore, detection methods need to have a LOD much lower than these threshold levels to ensure consumer safety and to confirm that even trace residual proteins are detected [56,57,58].

The research conducted by Westerhout et al. [59] comprehensively examines the methodology for determining individual threshold doses for various food allergens. Clinical provocation tests were used to establish these thresholds, revealing significant variability in individual threshold doses within the allergic population. The minimum doses that trigger allergic reactions vary depending on the type of allergen and the patient’s sensitivity. In 2021, maximum threshold values were established for eleven allergens, as outlined in a document from the Food and Agriculture Organization of the United Nations (FAO) and the World Health Organization (WHO) [59,60,61,62].

Table 2 presents the maximum thresholds for allergenic protein concentrations. Shrimp and lactose exhibit significantly higher concentration thresholds, indicating that larger amounts of these allergenic proteins are necessary to provoke an allergic reaction. In contrast, nuts and almonds require lower concentrations to induce allergic responses.

The data in Table 2 confirms the earlier statement that methods for detecting immunoreactive proteins must be extremely precise, with a detection limit significantly lower than the threshold values, to ensure consumer safety and enable the identification of even trace amounts of residual proteins.

## 3. Allergen-Reducing Food Processing

**Figure 2:**
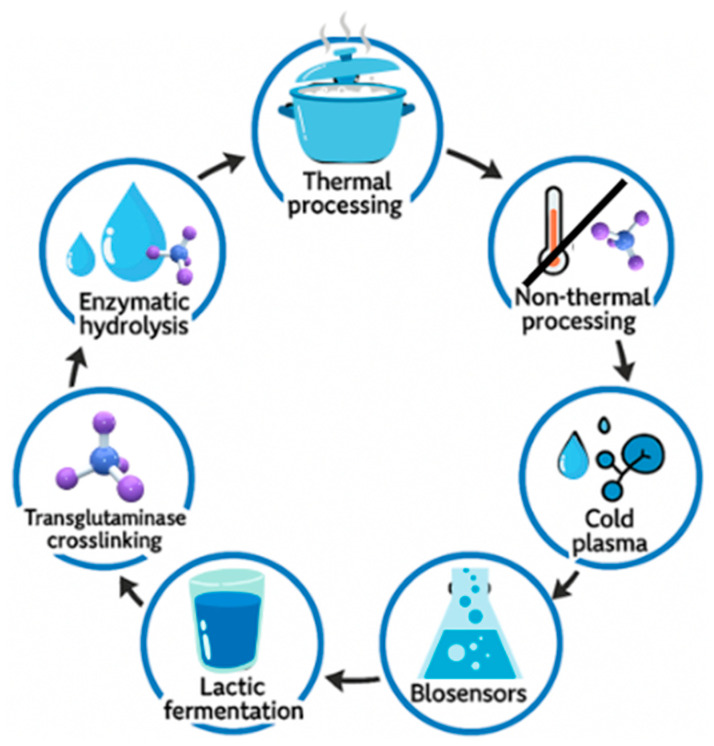
Food processing techniques are used to reduce the immunoreactivity of allergenic proteins. Drawing by authors with Canva software.

Reducing the immunoreactivity of allergenic proteins in food products is a crucial strategy for lowering the incidence of food allergies [62,63,64]. Therefore, suitable food processing methods offer a promising approach for reducing protein allergenicity. During food processing, changes in the secondary structure of proteins, caused by denaturation or degradation, affect their digestibility, bioavailability, and allergenic potential at the level of the host’s immune response [65,66]. Advanced food processing technologies with potential industrial applications include thermal and non-thermal treatments, enzymatic hydrolysis, cold plasma processing, lactic acid fermentation, and transglutaminase-mediated crosslinking [67,68,69] [Figure 2].

### 3.1. Thermal Food Processing

Thermal processing is the oldest and most widely used method for processing food products, aimed at improving the microbiological safety of the final product [70]. This process can reduce the allergenicity of food proteins through denaturation, aggregation, hydrolysis, and disruption of their interactions with other components, including carbohydrates and lipids [71,72,73,74,75,76,77]. The effects of heat treatment depend on temperature and exposure time and involve: (1) destabilization of the protein structure through the destruction of hydrogen and hydrophobic bonds (protein denaturation), resulting in decreased reactivity of the immune system; (2) partial denaturation of the protein leading to the formation of peptides and/or amino acids, which alters their digestion mechanisms and reduces the immune response [78,79]. For example, the disruption of the standard secondary structure of immunoreactive proteins occurs between 70 and 80 °C, the rearrangement of disulfide bonds occurs between 80 and 90 °C, and aggregation occurs between 90 and 100 °C [80,81,82,83]. Numerous studies have investigated the use of heat treatment to reduce the immunoreactivity of proteins or remove allergenic proteins in food products. Lechevalier et al. [84] used dry heat treatment to lessen the immunoreactivity of allergenic proteins in egg whites. Their study involved cooking at 100 °C for 15 min, targeting the main allergenic proteins, Gal d1 and Gal d2. This treatment induced partial structural changes in the protein, resulting in rod-shaped, unfolded aggregates with decreased immunoreactivity. Sudha et al. [85] reported that wet heat treatment has a positive impact on the immunogenicity of whole grain flour. The flour was steamed for 30 min and then dried for 2 h at 60 °C, resulting in a 41% reduction in the immunogenicity associated with the immunoreactive protein gliadin. Villa et al. [86] examined the effectiveness of baking, boiling, and autoclaving in reducing the immunoreactivity of lupin allergenic proteins. Results indicated that the immunoreactivity of lupin gamma-conglutinin allergenic proteins remained after baking at 180 °C, whereas notable protein fragmentation was observed following autoclaving at 138 °C for 20 min. Similarly, Ravinfran et al. [87] demonstrated that cooking decreased the allergenicity of soy protein by approximately 70–90%. Subsequently, Tobajas et al. [88] reported that heat treatment combined with ultrasound reduced the immunoreactivity of the peach allergenic protein Pru p3. Although heat treatment effectively reduces a product’s allergenic properties, it is essential to recognize that high temperatures can unmask or generate new epitopes [89].

One of the key mechanisms leading to the formation of neoantigens is the Maillard reaction, a non-enzymatic reaction between protein amino groups and reducing sugars [90,91]. This reaction produces advanced glycation end products, which can alter the structure of certain amino acids (cysteine, lysine, and arginine) [64,65,66,67,68,69,70]. These altered structures can be recognized by IgE antibodies, which are interpreted as an indication of an allergic reaction. An example of this is that when milk is heated, β-lactoglobulin undergoes a change, creating neoantigens that can trigger an immune response [92]. Similar changes can be observed in egg white (OVA) or nuts (Ara h 1) [93,94]. Furthermore, elevated temperatures can detrimentally affect the nutritional composition of products, which restricts the applicability of this method in meeting consumer requirements.

Another important aspect is the content of fats, carbohydrates, and sugars in the product, which remains unchanged due to heat treatment, allowing for analysis in a completely purified matrix. Consequently, when determining the level of protein immunoreactivity, these factors may affect the analysis result, leading to its falsification in nuts [93,94,95] or milk [96]. It could be explained by the aggregation of proteins that can still activate the immune system or the inability to remove linear epitopes [97].

### 3.2. Acid Treatment Method

In the food industry, acid treatment is less commonly used to reduce food allergenicity than heat processing. The mechanism of acid treatment involves denaturing proteins by disrupting hydrogen bonds and hydrophobic interactions with hydrochloric or sulphuric acid. This process leads to hydrolysis and fragmentation into smaller peptides, which may decrease their immunogenic potential. For example, Szymkiewicz et al. [98] examined the effect of cooking and enzymatic modifications on the allergenic properties of pea proteins. Their results indicated that hydrolysis with trypsin was more effective in reducing the allergenicity of pea proteins than alcalase. Research by Lee et al. [99] on the effect of acid treatment on peanut allergenicity (Ara h 1, Ara h 2) demonstrated that this method can effectively decrease protein immunoreactivity. Gel electrophoresis confirmed this; no protein bands were found in the acid-treated samples. Additionally, this relationship was verified using immunoblotting with rabbit antibodies. It was found that the antigenicity of Ara h 1 and Ara h 2 in peanuts treated with acetic acid at pH 2 was entirely undetectable. Similar studies on peanuts were conducted by Chung et al. [100], which demonstrated that treating peanuts (Ara h 1, Ara h 2) with a citric-phosphoric acid solution at pH 3 significantly reduces the allergen content. Comparable effects were also observed with oleic acid, which reduced the allergenicity of peanuts by approximately 40% [101].

Zhang et al. [102] revealed that the primary allergenic protein of oysters, Cra g1, did not break down under acidic conditions. Although acid treatment may appear to be a simple and effective method for reducing protein immunoreactivity, its lack of selectivity, the loss of protein functionality, and most importantly, the formation of by-products makes this approach unsafe.

Compared to other methods such as thermal processing, fermentation, or HPP, acid treatment is only a preliminary step in research [103,104,105]. Moreover, to fully verify and identify the epitopes most resistant to acid degradation, it is necessary to map them after acid treatment using the LC-MS/MS technique. This will facilitate the planning of targeted hydrolysis, enabling the selective removal of key epitopes responsible for allergic reactions [106]. While this method may reduce the immunoreactivity of proteins, it also impacts their biological properties. Additionally, it can lead to the formation of by-products, such as cysteine, which may create disulfides with an unpleasant odor. Acid treatment can also address issues related to pH restoration, waste disposal, and the high costs associated with acid neutralization. Implementing this technique in the food industry requires continuous monitoring of processing time and temperature, as deviations from these parameters can result in insufficient reduction in allergenic proteins’ immunoreactivity.

### 3.3. Microwave Treatment

The primary mechanism of this method involves generating microwave energy, which increases the temperature of the food by vibrating the water molecules within the matrix, ultimately leading to the inactivation or complete denaturation of the allergenic protein [107,108]. The effectiveness of this method depends on the type of allergenic protein, the duration and intensity of microwave treatment, as well as other processing factors [109,110,111,112,113]. It is essential to recognize that microwave treatment can affect the chemical structure and sensory properties of food products, which is vital for the sensory quality of the final product [109,110,111,112,113,114,115].

In the food industry, microwave treatment has been used to reduce allergenic protein content in kiwi [116]. This microwave treatment lowered the IgE-binding capacity of Act d 2 while increasing total antioxidant activity, digestibility, and peptide content. Wang et al. [117] reported that microwave pretreatment at 400 W for 20 s significantly decreased the allergenicity of ovalbumin by promoting hydrolysis and denaturation of IgE-binding epitopes. Furthermore, Meng et al. [118] confirmed that a 30-s exposure to 560 W microwaves significantly reduced the allergenicity of ovalbumin. The study highlighted that microwaves disrupted the protein’s structure, making it more accessible to alkaline protease. Conversely, Gazikalovic et al. [119] investigated the use of microwave pre-processing to reduce the immunoreactivity of wheat gluten proteins and demonstrated that, at a temperature of 100 °C and a power of 200 W, the immunoreactivity of gluten decreased by almost 2.5 times.

The use of microwave treatment in combination with other techniques, such as enzymatic hydrolysis, can significantly reduce IgE binding levels, indicating a decrease in protein immunoreactivity [120]. An example of such research is the work of Mecherfi et al., who assessed the impact of microwave treatment combined with enzymatic hydrolysis using pepsin on the allergenicity of the main cow’s milk protein, β-lactoglobulin. The results revealed that hydrolysis proceeded much faster after just 3 min of exposure to 200 W microwaves. Up to 56% of the protein was broken down, representing a much higher level of degradation compared to conventional heating. These findings were confirmed by SDS-PAGE electrophoresis and densitometric analysis of electrophoretic bands [121]. The synergistic effect of these methods promotes a deeper breakdown of the epitope structures responsible for allergic reactions, thereby enhancing the effectiveness of the process for reducing allergenicity [120,121,122].

One of the main disadvantages of microwave treatment is uneven heating and difficulty controlling the process due to the non-uniform microwave field. This leads to inconsistent degradation of immunoreactive proteins, and the effectiveness of reducing immunoreactivity may vary in different parts of the sample [123]. Furthermore, this technique often causes denaturation and aggregation of proteins that may be difficult to dissolve, hindering analysis methods such as ELISA, WB, or LC-MS/MS [120,121,122,123,124]. Although several studies have highlighted the potential benefits of using microwaves to preserve the nutritional quality of products, further research is needed to confirm their effectiveness in reducing the immunoreactivity of food proteins [116,117,118,119,120,121,122,123,124,125,126,127,128].

### 3.4. Food Fermentation

Microbiological fermentation is used in households and the food industry [129]. Fermented products are popular with consumers not only for their health benefits but also for their flavor [130]. During lactic fermentation, various aromatic compounds and organic acids are produced, contributing to the characteristic flavor notes of fermented products. These notes can include fresh, fruity, buttery, and even distinctly sour accents, shaping the unique sensory profile of the final product.

Fermentation helps extend the shelf life of food by inhibiting saprophytic microflora and improving digestibility [130,131,132,133,134]. The fermentation process is relatively straightforward, requires minimal labor, and involves low operating costs. Microbial fermentation is an anaerobic biochemical process in which lactic acid bacteria (LAB) convert sugars into lactic acid, a widely used chemical raw material in the food and pharmaceutical sectors [135,136]. LAB can be classified as homofermentative, producing mainly lactic acid, or heterofermentative, producing additional byproducts such as acetic acid, ethanol, and carbon dioxide [137]. They can create both L (+) and D (−) forms of lactic acid, or a mixture of the two isomers [138]. When selecting LAB strains, it is crucial to consider those with proteolytic enzyme activity, as these enzymes can hydrolyze proteins and reduce the immunoreactivity of allergenic proteins by disrupting their structure and lowering their molecular weight (kDa), ultimately diminishing their immunoreactivity [139,140].

Shimada et al. [141] demonstrated that fermenting Natto-A soybeans with *Bacillus subtilis* effectively degrades the allergenic protein Gly m 8, indicating the transformative potential of microbial fermentation in reducing allergenicity. Analysis by mass spectrometry (LC-MS/MS) indicates strong activity in degrading soy allergens, despite the detection of some limited degradation of the main soy protein, Gly m 8. The effect of fermentation using *Bacillus natto* on reducing the allergenicity of raw peanut pulp protein has been reported by Pi et al. [142]. The results showed that after 48 h, the immunoreactivity of peanut proteins was reduced by more than 77.3% and was positively correlated with the content of free sulfhydryl group. This means that some of the disulfide bonds have been reduced, leading to protein unfolding and loss of the native conformation of IgE epitopes. As a result, the ability of these proteins to bind IgE antibodies is reduced, and thus their immunoreactivity is decreased. Mecherfi et al. [143] screened 17 LAB strains for their proteolytic activity on gluten proteins and α-amylase inhibitors (ATIs). The results showed that the main gluten allergenic proteins were degraded after the fermentation of gluten by *Lactococcus lactis* LLGKC18. A significant decrease in gliadins, glutenins, and ATI antigenicity was observed after fermentation of gluten by this strain. Additionally, Schlegel et al. [144] reported that a combination of enzymatic hydrolysis (papain, alcalase 2.4 L, and pepsin) and lactic acid fermentation (*Lactobacillus sakei* ssp. carnosus, *Lactobacillus amylolyticus*, and *Lactobacillus helveticus*) effectively reduced lupin proteins to less than 0.5%. SDS-PAGE analysis and bead assays confirmed that the major allergen Lup an 1 was reduced to residual levels, significantly decreasing the immunoreactivity of allergenic proteins during this process. Song et al. [145] investigated the reduction in immunoreactivity through natural and induced soybean meal (SBM) fermentation using *Lactobacillus plantarum*, *Bifidobacterium lactis*, and *Saccharomyces cerevisiae*. Their findings showed that fermentation effectively lowered the immunoreactivity of soy proteins, especially those smaller than 20 kDa. Among the various treatments, *S. cerevisiae* and naturally fermented soybeans demonstrated the most significant reductions in IgE immunoreactivity, achieving decreases of up to 89% and 88%, respectively, compared to pooled human plasma.

However, it is worth noting that the fermentation process also has its disadvantages. Factors such as temperature, pH, and fermentation duration can significantly influence the success of reducing immunoreactivity in allergenic proteins. The selection of appropriate LAB strains is a crucial factor in determining the effectiveness of lactic fermentation in reducing food allergenicity [141,142,143]. Certain LAB strains demonstrate limited proteolytic capacity towards highly structured proteins, which may result in incomplete degradation and a residual presence of immunoreactive protein fractions. Furthermore, protein hydrolysis during fermentation produces a complex mixture of peptides that vary in length, conformation, and amino acid composition, some of which may retain or even develop IgE-binding capacity. The formation of new peptides with potential allergenic properties complicates the clear assessment of the safety of fermented products [141,142,143,144,145]. From a technological perspective, extensive protein hydrolysis may also alter the rheological properties of the product, affecting its texture, viscosity, and overall stability due to changes in the colloidal structure of the system [142,143,144,145,146]. These factors collectively hinder the full standardization of lactic fermentation processes, making it challenging to achieve consistent allergen reduction and reproducible results across different production batches.

### 3.5. High-Pressure Processing (HPP)

High-pressure processing (HPP) is a modern food preservation technique that effectively maintains freshness, nutritional content, and natural flavor and aroma while extending the product’s shelf life [147]. HPP involves applying high hydrostatic pressure (ranging from 100 MPa to 600 MPa) at temperatures below 100 °C, which inactivates microorganisms and enzymes responsible for food degradation, such as polyphenol oxidase and lipase [148,149,150]. This approach minimizes the loss of heat-sensitive components, such as vitamin C, carotenoids, and polyphenols [151,152,153,154,155]. The impact of the HPP process on food’s chemical composition and sensory attributes depends on several factors, including the pressure level, processing duration, and the specific characteristics of the raw material.

The HPP method utilizes high pressure to alter protein structures without breaking covalent bonds, thereby preserving the fundamental biological value of proteins while eliminating some conformational epitopes responsible for IgE reactions [155,156,157,158]. Additionally, this method can be applied to a wide range of products, from liquids to solids, making it a technologically adaptable approach. Its versatile nature enables it to be combined with other techniques for reducing immunoreactive proteins, such as acid or enzymatic treatments. However, despite its many advantages, the HPP method also faces some limitations, including equipment costs and the need for appropriate packaging.

Numerous studies have suggested the potential use of HPP to reduce the allergenicity of various food products, including legumes, grains, seafood, meat, dairy products, fruits, and vegetables [159,160,161,162]. HPP technology can alter the allergenicity of proteins through several mechanisms, including denaturation, aggregation, and conformational changes, thereby reducing their allergenic potential [163,164].

The selection of pressure is crucial because its value affects the balance between different conformational states of the protein, leading to the breaking of weak non-covalent bonds, which results in partial or complete unfolding of the protein structure. Unfortunately, this process may be reversible after the pressure is lowered. Furthermore, the exposure of certain fragments of the amino acid sequence may increase the availability of epitopes recognized by IgE antibodies, potentially exacerbating the allergic response. On the other hand, unfolding of proteins and dissociation of their subunits can lead to the destruction of conformational epitopes, the recognition of which requires an intact spatial structure, resulting in reduced immunoreactivity. Therefore, it is worth emphasizing that the effect of HPP on immunoreactive proteins is complex and depends on the type of epitopes; in some cases, it may lead to a reduction in allergenicity, while in others it may maintain or even increase it. It is also worth noting that changes in protein conformation can modify existing epitopes, which in turn can result in their non-recognition by IgE antibodies, although they may still be allergenic [165,166].

The size of a protein molecule also plays a crucial role in determining its immunoreactivity. Large proteins containing many subunits are characterized by weaker non-covalent bonds, which makes them more susceptible to dissociation and unfolding. This process can lead to the destruction of conformational epitopes. Smaller proteins, on the other hand, may be more resistant to denaturation, retaining some of their immunoreactivity. The second important factor is disulfide bonds, which stabilize the tertiary and quaternary structure of proteins. Proteins rich in disulfide bridges show much greater resistance to unfolding under high pressure, as covalent bonds maintain the integrity of their structure.

Numerous scientific studies confirm the validity of these observations. A study by Pan et al. [167] demonstrated that HPP (600 MPa for 1200 s) alters the spatial structure of the main immunoreactive allergenic protein in peanuts, Ara h1, resulting in a 74.32% reduction in immunoreactivity. On the other hand, HPP can be synergistically combined with different techniques to mitigate the immunoreactivity of allergenic proteins more effectively. Landim et al. [168] demonstrated that combining HPP (100, 250, and 400 MPa) with alternative proteases, Novo Pro-D^®^ (NPD, Sigma-Aldrich, St. Louis, MO, USA), effectively reduced the allergenicity of whey protein concentrate (WPC), with HPP used as a pretreatment step for WPC. Pre-treatment of WPC using HPP enhanced the in vitro antioxidant capacity and resulted in a 100% reduction in immunoreactivity to β-lactoglobulin in a shorter processing time. Similarly, Lozano-Ojalvo et al. [169] showed that processing whey proteins with pepsin under HPP (400 MPa/30 min) impacted the immunoreactivity of whey proteins by hydrolysis into peptides with molecular weights in the range of 10 to 3 kDa.

### 3.6. Ultrasound, Pulsed Electric Field (PEF)

Ultrasound technology utilizes high-frequency sound waves, typically above 20 kHz, to apply vibrations to food products through a water medium [170]. Exposing a food sample to ultrasound causes acoustic cavitation, characterized by the formation and collapse of microbubbles, which results in significant changes to the sample’s structure. Acoustic cavitation can influence the physical and chemical properties of food, including the denaturation of proteins, which is essential for reducing food allergenicity [171]. Ultrasound treatment may lead to alterations in the secondary/tertiary structures of allergenic proteins and the development of intra- or intermolecular interactions that could affect their allergenic potential [172,173]. This method is most effective in the case of conformational epitopes, while its effectiveness in relation to linear epitopes (Ara h 1, Gly m 5, or Bos d 8) is limited; often, only a partial reduction in immunoreactivity is observed, rather than complete neutralization of the allergen [174]. However, only a limited number of studies have examined the impact of ultrasound treatment on food allergens from either animal or plant sources, and the findings of these studies have been inconsistent [175,176,177,178]. Tobajas et al. [88] evaluated the impact of sequential microwave heating (140 °C for 30 min) followed by ultrasound treatment (26 kHz, 150 W for 30 min) on the denaturation and allergenicity of extracts from peach peel and pulp. Results showed that combining these treatments did not reduce the binding affinity of Pru p 3 to rabbit-specific IgG or human IgE from the sera of patients with peach allergies. Furthermore, Long et al. [179] reported that ultrasound treatment for 60 min at 300 W and 55 °C, at 59 kHz, significantly reduced Amandin’s immunoreactivity by 15.61%. The effectiveness of ultrasound in combination with processes such as heating, glycation, germination, hydrolysis, fermentation, irradiation, and polyphenol treatment for reducing protein allergenicity from peanuts, seafood, fish, eggs, soy, milk, and wheat has also been documented by Pi et al. [180]. The authors highlighted that ultrasound causes structural modifications and even degradation of proteins, primarily through cavitation effects, leading to reduced allergenicity. Additionally, Li et al. [181] reported that high-intensity ultrasound could decrease the allergenicity of the tropomyosin fraction of shrimp proteins, which are responsible for allergic reactions. In summary, the effectiveness of ultrasound as a standalone method depends on several variables, including ultrasound power, exposure time, frequency, and the type of food being processed. Furthermore, the duration of ultrasound application and the conditions under which ultrasound-assisted technologies are employed significantly influence their efficacy [173,174,175,176,177,178,179,180,181,182,183].

### 3.7. UV Radiation

Exposure to ultraviolet radiation in the UV range (200–280 nm) can cause the denaturation of allergen proteins in foodstuffs by breaking down their secondary structures [181,182,183,184,185,186,187,188,189], resulting in a decrease in their immunoreactivity. Yang et al. [190] showed that pulsed UV light effectively deactivated Ara h 2 in peanut butter. Additionally, they found that UV irradiation significantly reduced allergenicity in soybean extract by lowering soy allergens, such as glycine and β-conglycine, by 20%, 44%, and 50% after exposure for 2, 4, and 6 min, respectively. Salazar et al. [191] reported that pulsed UV radiation notably reduced the immunoreactivity of casein and β-lactoglobulin in milk. Their study investigated the effects of different pulsed UV light fluences (ranging from 0.1 to 10 J/cm^2^) on commercial cow’s milk proteins, including casein, α-lactalbumin, and β-lactoglobulin. Using ELISA, they observed that immunoreactivity levels for casein and β-lactoglobulin decreased by 24% and 47% at a UV dose of 10 J/cm^2^. Furthermore, Mel et al. [192] demonstrated that radiation treatment of crab meat effectively diminishes its immunoreactivity. This study applied an electron beam to crab meat at doses ranging from 1 to 9 kGy, followed by tests to assess its IgG-binding capacity. A similar study on shrimp meat was carried out by Guan et al. [193], who applied electron beam irradiation at doses ranging from 0 to 9 kGy, with the most significant reduction in IgG binding (59%) observed at 7 kGy.

It should be noted that this process is technically difficult because food does not allow UV rays to pass through. In the case of foods with a heterogeneous structure, most of the UV energy is absorbed in the surface layers, resulting in minimal protein denaturation within the sample [194,195]. Another disadvantage of this method is the induction of photooxidation of aromatic amino acids and the formation of reactive oxygen species (ROS), which can lead to the generation of neoantigens [194,195,196]. In addition, the difficulty in standardizing and controlling the process does not bode well for this method. This method does not significantly reduce the content of allergenic proteins on its own, but it can be used as a preliminary method for further techniques to reduce the immunoreactivity of proteins in food products.

### 3.8. Enzymatic Modification

Enzymatic hydrolysis (proteolysis) is a well-established non-thermal processing technique used to reduce the allergenicity of various food allergens [197,198,199]. This method disrupts the protein structure, altering the linear and conformational epitopes of food allergens [200]. However, its effect on allergenicity depends on the amino acid sequence and the secondary structure of the protein. Additionally, selecting suitable enzymes and conditions, such as temperature, pH, and reaction time, is crucial for effectively reducing the protein’s immunoreactivity in the final product [201]. Enzymes like trypsin, pepsin, and chymotrypsin are commonly employed for hydrolysis, while other enzymes of plant and microbial origin, including papain, alcalase, and pronase, have also been utilized [202]. Numerous studies have demonstrated the effectiveness of enzymatic hydrolysis in decreasing the allergenicity of food products [173,174,175,176,177,178,179,180]. Enzymatic hydrolysis has successfully reduced allergenicity in food allergens derived from milk, eggs, lentils, soybeans, chickpeas, and peanuts [203,204,205,206,207,208,209,210,211,212,213].

For example, Calcinai et al. [214] reported that treatment with alcalase and papain reduced the immunoreactivity of peas, chickpeas, and white beans. Alcalase demonstrated the highest effectiveness in lowering immunoreactivity in chickpeas and green peas. In contrast, the papain enzyme was found to be less effective. Cabanillas et al. [215] showed that treatment of peanut flour with alcalase or the combination of alcalase and flavourzyme significantly reduced IgE-binding of the protein extract. A study on cow’s milk proteins subjected to enzymatic hydrolysis found that the allergic response was decreased by approximately 50%. This reduction was validated using three enzymes: alcalase, protamex, and flavourzyme [215]. Damodaran et al. [216] demonstrated that a two-step enzymatic approach effectively reduced the immunoreactivity of whey protein and casein. The first step involved partial hydrolysis of the proteins using proteases, while the second step utilized microbial-derived transglutaminase for repolymerization. The findings showed that partial hydrolysis of whey protein isolates using chymotrypsin, trypsin, or thermolysin preserved approximately 80%, 30%, and 20% of the original immunoreactivity. Subsequently, transglutaminase polymerization significantly decreased immunoreactivity to 45%, 35%, and 5% of the original levels, respectively. In the case of casein, the combination of hydrolysis and repolymerization nearly eliminated immunoreactivity, marking a significant advancement compared to the native form of this protein.

Enzymatic modification also presents several significant technological and immunological challenges. Research by Pang et al. [217] showed that some peptides produced by enzymes can retain epitopes recognized by IgE antibodies. Another study [218] demonstrated that newly formed epitopes after enzymatic treatment are more immunoreactive than the parent proteins, which may be attributed to a change in their conformation. An additional limitation may be incomplete hydrolysis, which makes it difficult to obtain reproducible results and thus complicates the standardization of the process.

An additional disadvantage of this method is the limited access of enzymes to coiled protein structures or disulfide bridges, which may result in some epitopes remaining intact and thus maintaining the residual immunoreactivity of the product. Another technological challenge may be the impact of enzymes on reducing protein solubility, which can adversely affect the texture and stability of products [219,220].

### 3.9. Cold Plasma

Cold plasma is an innovative, non-thermal food processing technology that is gaining increasing use in the food industry [221,222]. This process enhances properties by effectively inactivating food-spoiling microflora and minimizing the risk of lowering food’s nutritional and sensory values without using high temperatures [223,224,225]. Plasma is generated through various energy sources capable of ionizing gases, including electrical, thermal, optical, and electromagnetic sources, as well as radioactive and X-ray sources, which produce a mixture of electrons, ions, and neutral molecules [226,227]. It has been reported that cold plasma can modify proteins, affecting their conformational structure and reducing their allergenic potential while preserving the taste and quality of food [228,229]. This technology can be utilized across various industries, providing effective disinfection without the risk of chemical contamination [230,231].

Hsu et al. [232] applied cold argon plasma to reduce the allergenicity of the Ara h 1 protein, a major allergen in peanuts. Results showed that after 15 min of exposure, the immunoreactivity of Ara h 1 was reduced by 66%. Similarly, Hsieh [233] observed decreases in Ara h 1 by 91% and 76% after 30 min of exposure to air and nitrogen plasma, respectively. The authors noted that air plasma treatment led to more noticeable structural changes and protein aggregation of Ara h 1 than nitrogen plasma. Venkataratnam et al. [234] demonstrated that cold plasma treatment significantly reduced the allergenicity of the peanut allergens Ara h 1 and Ara h 2 using pin-plate atmospheric plasma at various processing times. This technique decreased the solubility of the proteins and altered their structure; prolonged exposure to cold plasma correlated with reduced solubility for both Ara h 1 and Ara h 2. ELISA results indicated a 65% reduction in allergenic potential for Ara h 1 and a 66% reduction for Ara h 2. Using cold plasma, Chen et al. [235] investigated the reduction in coconut globulin in coconut milk. Results showed that this process altered the protein structure, decreasing the affinity between coconut globulin and IgE by reducing the constant association rate (Ka) from 2.17 × 10^4^/M to 0.64 × 10^4^/M, thereby diminishing IgE-dependent allergic reactions. Zhang et al. [236] demonstrated that cold plasma treatment effectively influences the immunoreactivity of egg allergenic proteins. In their study, egg white was exposed to cold plasma for 30 min, resulting in a nearly 20% reduction in IgG binding capacity. Additionally, the digestibility of egg white improved by 140%, while its foaming and emulsifying capacities increased by approximately 8% [236]. Tropomyosin (TM) is the predominant allergen found in crustaceans. Zou et al. [237] utilized cold plasma with glycation treatment on the processing and cleavage sites of TM trypsin from shrimp. A 20% reduction in IgE binding capacity was observed compared to the original control product.

The cold plasma method also has its drawbacks. Prolonged exposure can cause undesirable changes in the color, smell, and taste of the product. The oxidation of lipids or aromatic amino acids may be responsible for this phenomenon. Additionally, it can impact solubility and emulsifying ability through the oxidation and fragmentation of polypeptide chains. The effectiveness of this method depends on many variables (type of gas, discharge intensity, voltage, frequency, exposure time), which makes it difficult to standardize. The most significant disadvantage of this method in terms of immunoreactivity is the potential to create neoantigens. Oxidative reactions can modify amino acid residues in a way that leads to the formation of new epitopes. In addition, active plasma particles only affect the surface of the material, so in dense products, the effect of allergenicity reduction only affects the outer layers, while the interior remains virtually untouched.

The advantages and disadvantages of various food processing technologies used to decrease allergenicity of proteins in food products are presented in Table 3.

Technological methods aimed at reducing the immunoreactivity of allergenic proteins are a vital focus of research for enhancing food safety. Current strategies not only seek to limit the ability of proteins to trigger an immunoreactive response but also aim to maintain their nutritional and functional properties. Each technique employed to diminish immunoreactivity uniquely affects the protein’s structure, either by changing its spatial conformation or by destroying epitopes. Consequently, proteins lose their ability to bind to IgE antibodies, resulting in a decrease in their allergenic potential. This highlights the importance of an integrated approach, where allergenic protein modification processes are closely tied to methods for precise quantification and qualitative assessment. Only through simultaneous analysis of structural and immunological changes can an accurate evaluation of the true reduction in immunoreactive proteins be achieved.

## 4. Methods for the Detection of Allergenic Proteins

Various direct and indirect methods have been developed to detect immunoreactive proteins. Several immunochemical techniques serve as direct methods for identifying allergens, including enzyme-linked immunosorbent assay (ELISA) and immunoblotting (WB) [260,261]. However, genetic techniques such as polymerase chain reaction (PCR) enable the indirect detection of specific DNA fragments that encode allergenic proteins [262,263,264]. Additionally, analytical methods such as LC-MS/MS, NRM, or biosensors play a crucial role in the comprehensive analysis of allergenic proteins, enabling their precise identification, structural characterization, and quantification, even within complex food matrices.

**Figure 3:**
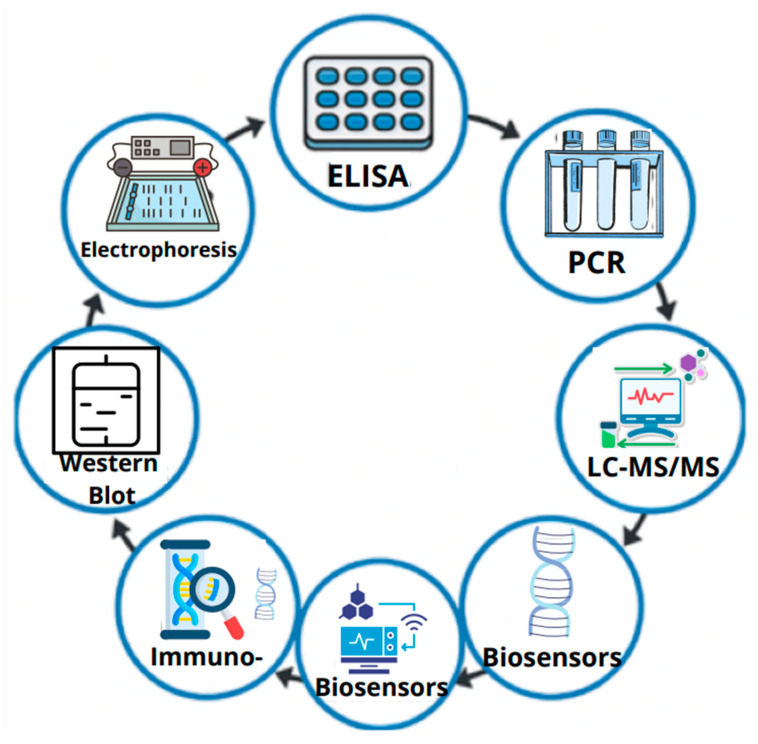
Techniques for determining immunoreactive proteins in food. Drawing by authors using Canva software.

Figure 3 illustrates examples of techniques for identifying proteins with immunoreactive potential.

### 4.1. Immunoblotting

#### 4.1.1. Enzyme-Linked Immunosorbent Assay (ELISA)

Enzyme-Linked Immunosorbent Assay (ELISA) is commonly used to identify food allergenic proteins [265,266,267,268]. This quantitative technique employs specific antibodies to accurately measure the presence and concentration of allergens [269,270]. The ELISA method involves immobilizing an antibody or antigen on a microtiter plate and adding an enzyme-linked antibody that binds specifically to the target antigen [271,272,273,274,275,276,277]. It is most frequently utilized in routine food quality control due to its specificity, simplicity, low cost, and rapid analysis time [265,266,267,268,269,270,271,272,273,274,275,276,277,278]. Zhu et al. [279] employed ELISA to validate the detection of soy allergenic proteins using rabbit polyclonal antibodies and goat antibodies against soy proteins. Yayla et al. [280] applied ELISA to detect A1 and A2 β-casein proteins in cow’s milk. Dasanayaka et al. [281] employed ELISA to evaluate the effects of various food processing methods, including physical and chemical treatments, finding that thermal processing significantly altered the structure and chemical properties of fish proteins. Shen et al. [282] developed an ELISA to detect the Ana o3 protein in cashew nuts, utilizing two monoclonal antibodies (2E10 and 5D7). The assay demonstrated high specificity and sensitivity, with a detection limit of 0.6 ng/mL. Civera et al. [283] employed amandin antibodies labeled with horseradish peroxidase to detect the allergenic Pru du 6 protein of almond, achieving a detection level of 2 ng/mL. Several studies have focused on developing sensitive and specific sandwich ELISAs for detecting allergenic proteins in food products [284,285,286].

Due to its limited sensitivity, the ELISA method faces certain restrictions in detecting allergen-specific IgE antibodies [287]. ELISA can only detect IgE antibodies at relatively high levels of sensitization; therefore, in clinical practice, automated analyzers or alternative technologies, such as ImmunoCAP, Immulite, or microarray-based assays, are more frequently utilized because they provide greater accuracy and sensitivity [287,288,289]. Another limitation of ELISA is its dependence on the structural integrity of epitopes [288,289]. Food processing procedures can alter or degrade epitopes, thereby reducing antibody recognition and potentially leading to false-negative results [290]. Additionally, ELISA requires the development of individual assays for each specific allergen. Commercial ELISA kits also vary in the types of antibodies used, as well as their sensitivity and detection ranges, which makes it difficult to compare results across different laboratories.

#### 4.1.2. SDS-PAGE: Separation of Proteins by Electrophoresis in a Polyacrylamide Gel

SDS-PAGE is a technique used to separate molecules based on their electrical charge and size. When combined with 2D-E, it becomes a valuable method for detecting and isolating allergenic proteins from various food matrices [291,292]. In SDS-PAGE, proteins are separated by molecular weight because SDS denatures them and imparts a uniform negative charge. Detection through dyes such as Coomassie Blue allows for quantification and visualization [293,294,295,296]. In 2D-E, proteins are separated according to their isoelectric points and by their relative molecular weights using sodium dodecyl sulfate- polyacrylamide gel electrophoresis (SDS-PAGE) [297,298,299]. A study by Zienkiewicz et al. [300] utilized 2D-E to identify allergenic proteins, specifically Ole e 1, Ole e 2, and Ole e 5, in olive pollen. Paired with 2D-E blotting, fluorescent probes effectively eliminated signal interference, enabling the accurate quantification of different allergenic protein isoforms. Another study by Mendez et al. [301] utilized 2D-E combined with LC-MS/MS analysis to detect and characterize carbonylated proteins within a complex mixture from various fish tissues. De Silva et al. [302] also employed SDS-PAGE and LC-MS/MS to identify food allergens in the serum of individuals allergic to egg yolk. SDS-PAGE is often combined with Western blot (WB) analysis. In this coupled approach, proteins separated by SDS-PAGE are transferred onto a membrane and subsequently identified using specific antibodies, allowing for precise detection of target allergenic proteins.

Like any analytical technique, SDS-PAGE has certain limitations, particularly when applied to proteins that undergo technological processing, such as heating, fermentation, or high-pressure treatment [297]. During food processing, Maillard reactions, intermolecular cross-linking, or oxidation may occur, which can alter the molecular weight of proteins, resulting in the formation of heterogeneous complexes with unpredictable migration patterns in the gel [297,298,299,300,301,302,303]. Moreover, the disadvantage of this method is the difficulty in separating proteins with similar molecular weights, as well as the incorrect migration of proteins if they are too large or too small. In addition, contaminants such as lipids or salts can interfere with protein migration on the gel [304,305,306,307].

#### 4.1.3. Western Blot (WB) Analysis

WB is a common technique for detecting proteins, based on their molecular weight and specific antibody interactions [308,309]. In this method, antigens are separated by their molecular weight using SDS-PAGE and then transferred onto a polyvinylidene difluoride or nitrocellulose membrane, which acts as the solid phase for the antigen–antibody reaction [310,311]. Secondary antibodies are detected using fluorescence, colorimetric assays, or chemiluminescent assays, which produce visible bands on the membrane that indicate the presence of targeted proteins [311,312]. Western blotting (WB) has been widely used for the detection and characterization of immunoreactive proteins, as shown by numerous studies emphasizing its reliability and specificity in identifying allergenic and antigenic protein fractions [310,311,312,313,314,315,316,317,318,319].

Using WB, Pfeil [313] determined the water-soluble allergens of wheat pasta, including Tri v Bd 47, Tri v Bd 17, and Tri v Bd 15, which correspond to molecular weights of 47, 17, and 15 kDa. Zhou et al. [314] used WB to identify the Tyr p 31 allergenic protein from *Tyrophagus putrescentiae*, a common mite that induces allergies. Western blot analysis revealed that the serum of children with allergic diseases exhibited a specific IgE response to rTyr p 31, with frequencies of 72.41% and 85.7%, respectively [314]. However, Anjos et al. [315] used WB to identify allergenic proteins in fish meat, including β-parvalbumin. The study focused on extracting three protein fractions from white fish muscle: the first fraction consisted of sarcoplasmic proteins, the second of myofibrillar proteins, and the third included insoluble components. Moreover, WB was also used to detect Ara h 1, Ara h 2, Ara h 3, and Ara h 6 proteins in peanuts [316]. WB has proven effective in identifying novel allergenic proteins and epitopes relevant for food allergy diagnosis and therapy. De Angelis et al. [317], analyzing sera from 14 hazelnut-allergic patients, characterized major hazelnut allergens including Cor a 2, Cor a 8, Cor a 9, and Cor a 11. Similarly, Iddagoda et al. [318] compared allergen profiles in fresh and cooked coconut milk, as well as unrefined coconut oil, produced using both wet and dry methods. Using sera from 18 hypersensitive patients, immunoblotting revealed six previously unreported coconut proteins (5–128 kDa) with allergenic potential. WB is frequently used in conjunction with other analytical techniques, such as SDS-PAGE or LC-MS/MS, to enhance the identification of allergens. For instance, Chen et al. [319] investigated almond vicilin by first separating proteins via SDS-PAGE (4–10% polyacrylamide gel), followed by WB analysis of excised peptide bands. Their results showed that 44% of the samples contained IgE antibodies recognizing recombinant almond vicilin, confirming its allergenic potential.

Despite its high specificity and analytical reliability, the Western blot (WB) technique has several limitations, especially when applied to processed food matrices such as dairy products, baked goods, meat products, or fermented foods [310,311,312]. A primary limitation is the method’s complete dependence on the specificity and affinity of the antibodies used in detection. Thermal processing, fermentation, or high-pressure treatment can cause protein denaturation, aggregation, or fragmentation, leading to the formation of small peptides that may not be efficiently transferred onto the membrane or recognized by antibodies [311,312,313]. Additionally, in complex food matrices rich in lipids, carbohydrates, or salts, protein extraction can be hindered, which reduces detection efficiency and potentially leads to false-negative results [312,313,314,315,316,317,318,319].

### 4.2. Mass Spectrometry-Based Techniques

#### 4.2.1. Liquid Chromatography Coupled with Tandem Mass Spectrometry (LC-MS/MS)

Liquid chromatography combined with tandem mass spectrometry (LC-MS/MS) is one of the most advanced tools used in modern proteomics for the identification and quantitative determination of proteins [320,321,322,323,324,325,326,327,328,329,330,331]. Unlike traditional methods, LC-MS/MS can concurrently identify and quantify multiple allergenic proteins within a single analysis. LC-MS/MS is a highly sensitive and specific method, comprising two stages [325,326,327,328,329,330,331,332]. The first stage involves separating components and segregating them based on their viscoelastic properties through liquid chromatography (LC). Subsequently, tandem mass spectrometry (MS/MS) is used to identify and quantify the selected proteins.

LC-MS/MS has been utilized to detect food allergens from milk, eggs, peanuts, and soy [285,286]. Grosch et al. [324], employing LC-MS/MS, determined the content of Pol d 5 protein in wasp venom. New et al. [327] used LC-MS to identify trypsin peptides in peanuts, eggs, skimmed milk, and soybeans, with a detection limit of 10 ppm. Sagu et al. [328] used LC-MS/MS to detect the corresponding molecular variants of the proteins: Pru du6.0101, Cor a 11.0101, Pis v 3.0101, and Jug r 6.0101 in peanuts. Several studies combined LC-MS/MS with SDS-PAGE as a preliminary step to prepare samples for further quantitative and qualitative analysis [329,330]. For example, Wang et al. [329] employed SDS-PAGE in conjunction with LC-MS/MS to elucidate the carbohydrate structures of glycoproteins from Ginkgo biloba seeds. Similarly, Babu et al. [330] reported methods using LC-MS/MS with SDS-PAGE to detect polyphenol oxidase (PPO) in eggplant. SDS-PAGE was employed to separate protein mixtures, while LC-MS/MS identified two PPO isoforms, PPO1 and PPO4. Consequently, PPO4 was classified as a new allergen based on the presence of an IgE epitope. Meanwhile, Torii et al. [331] used LC-MS/MS with MRM (Multiple Reaction Monitoring) to identify allergenic proteins in walnuts (Jug r 1 and Jug r 2) and almonds (Pru du 6) in processed foods. The application of LC-MS/MS to detect allergenic proteins derived from wheat (Tri a 36, Tri a 26, Tri a 20), eggs (Gal d 6, Gal d 4, Gal d 3), buckwheat (Fag e 1), milk (Bos d 9, Bos d 10, Bos d 5), crustaceans (Pen m 1), and peanuts (Ara h 1, Ara h 2, Ara h 3) has also been documented [330,331,332,333].

LC-MS/MS offers several benefits, including high sensitivity and specificity, as well as the ability to analyze multiple proteins simultaneously in a single measurement [320,321,322,323,324,325,326,327,328,329,330,331,332,333,334,335]. These benefits ensure dependable results, even for highly complex biological samples. However, this method also has drawbacks, including high equipment costs and the need for reliable sample preparation with high purity. Additionally, complex bioinformatic analysis of the data is often necessary [330,331,332]. Another challenge is selecting stable and unique peptide markers for a specific protein, which is complicated by trypsin digestion that produces many peptides, only some of which are suitable for detection and identification. Furthermore, changes in temperature, pressure, and pH during food processing can lead to peptide degradation or post-translational modifications, making it more challenging to detect markers and reducing the sensitivity and specificity of the analysis [320,321,322,323,324,325,326,327,328,329,330,331]. Moreover, the procedures and reference materials for analyzing proteins and peptides via LC-MS/MS are limited, as different conditions are used for digestion, protein extraction, instrument calibration, and data interpretation. The absence of certified reference materials for most marker peptides also complicates efforts to ensure data consistency.

#### 4.2.2. Nuclear Magnetic Resonance (NMR) Spectroscopy

Nuclear magnetic resonance (NMR) spectroscopy is employed in food quality control, authentication, nutritional research, and the analysis of food structures and reactions [336]. NMR enables a detailed examination of the structure, dynamics, and interactions of food proteins at the atomic level, making it a valuable technique for detecting allergenic proteins in food products [337,338,339]. Zeindl and Tollinger [340] utilized NMR to identify PR-10 family proteins associated with birch pollen-related pathogenesis. In a separate study, Zeindl et al. [341] used NMR to determine the three-dimensional structures of PR-10 allergens, such as Act c 8 and Act d 8, identified in two kiwi varieties (Actinidia chinensis and Actinidia deliciosa). Additionally, NMR was employed to analyze the Bet v 1 protein, a major birch pollen allergen associated with cross-reactivity to other allergens [342,343]. Similarly, Nishino et al. [344] applied NMR for the structural analysis of the Fra a 1.01 and Fra a 1.02 isoforms in strawberries following heat treatment. Alessandri et al. [345] classified allergenic proteins based on their tertiary structure characteristics using one-dimensional ^1^H-NMR spectroscopy (1D^1H-NMR^). The analysis included allergenic proteins from apples, peaches, hazelnuts, peanuts, caseins from cow’s and goat’s milk, and shrimp tropomyosin. The findings demonstrated that this method effectively evaluates epitope accessibility to IgE antibodies, providing structural insights into immunoreactive proteins. Hazebrouck et al. [346] demonstrated the use of NMR to characterize allergenic structural proteins. Their results showed that the immunogenicity of peanut allergens Ara h 2 and Ara h 6 mainly depends on the presence of conformational epitopes. In Ara h 2, a region spanning amino acid residues 33–81 contains both a major conformational and a linear epitope, whose close spatial positioning likely explains its strong immunogenicity and ability to trigger mast cell degranulation.

Nuclear magnetic resonance (NMR) spectroscopy has inherent limitations. One significant challenge is the need for large amounts of highly purified protein. Protein levels are often reduced in processed or extensively modified food samples because of interactions with other components or partial degradation during processing [336,337,338,339,340,341,342,343,344,345,346]. This makes it difficult or sometimes impossible to obtain samples with suitable concentrations for NMR analysis. Furthermore, NMR lacks the sensitivity to detect trace allergen levels, as it requires relatively high analyte concentrations for clear signal detection. Interpreting NMR spectra is also complicated, requiring advanced computational analysis and structural modeling, especially when analyzing large or heterogeneous molecules. Additionally, compounds such as polyphenols, sugars, and lipids can interfere with spectral signals, adding further complexity to data interpretation and necessitating more sophisticated analytical techniques.

### 4.3. Biological Biosensors

Biosensors are analytical devices that combine biological elements, such as antibodies, with a physical transducer, for example, an electrochemical transducer, allowing the detection of a specific analyte, including allergenic proteins [347,348,349,350,351,352,353,354,355,356,357,358,359,360]. They are also extensively used in allergy diagnostics because of their ability to quickly and selectively identify allergens in food through changes in electrode impedance and to measure the level of IgE proteins in the patient’s serum [350,351]. Biosensors offer a faster, more accessible, and sensitive alternative for detecting these proteins [352,353,354].

Jiang et al. [355] conducted research in which Fe_3_O_4_ nanoparticles were encapsulated within a SiO_2_ shell, creating fluorescent magnetic beads that were incorporated into liposomes. The resulting cationic magnetic fluorescent nanoparticles were utilized in magnetic transfection of mast cells and electrochemical detection of allergens, including the shrimp allergen Pen a 1 and the fish allergen PV. The developed method accelerated and improved the detection of food allergens after activation in response to the control reaction. Vasilescu et al. [356] employed biosensors and biotests to detect allergens in packaged foods, evaluating different biosensor types and their respective advantages and limitations. Their findings lay the groundwork for enhancing allergen detection techniques and adopting innovative methods in the food industry to better protect consumers. Xiao et al. [357] used an imaging surface plasmon resonance (iSPR) biosensor to detect hazelnut allergens in plant-based beverages. Their approach utilized an innovative iSPR platform featuring a 3D-printed microfluidic system integrated with smartphone-based optics. The method achieved detection limits ranging from 0.04 to 0.53 µg/mL, demonstrating high sensitivity and practical relevance for the analysis of food samples. However, Usher and Showalter et al. [358] reported that coupling biosensors with NMR facilitated the identification of otherwise inaccessible antigenic epitopes, such as the ART v3 protein from ragweed pollen. The approach relied on hydrogen–deuterium exchange NMR (HDX-NMR) under sub-stoichiometric antibody conditions, enabling the selective retention of protected sites and thus allowing precise epitope mapping.

Biosensors have demonstrated excellent analytical performance under controlled laboratory conditions; however, their transition to real-world applications requires careful consideration of their technology readiness level (TRL) [357,358,359]. Although these systems show great potential, most remain at the experimental or prototype stage and are not yet integrated into routine quality control procedures in the food industry or semi-industrial environments [359]. One of the main barriers to large-scale implementation is the limited stability of biological sensing elements, which are prone to denaturation, degradation, and loss of activity under industrial processing conditions. Furthermore, the standardization and reproducibility of biosensor fabrication present considerable challenges. While laboratory-based biosensors can achieve high sensitivity and selectivity, their adaptation to industrial settings is hindered by production costs, calibration requirements, and issues related to process standardization. Another critical limitation arises from matrix interferences in complex food systems [355,356,357,358]. Components of processed foods, particularly those subjected to denaturation, aggregation, or enzymatic hydrolysis, can distort analytical signals or hinder antigen–bioreceptor interactions [356,357,358,359]. Such structural alterations of proteins may obscure or modify allergenic epitopes, reducing recognition accuracy and potentially leading to false-negative results. Consequently, while biosensors represent a highly promising direction for rapid allergen detection, significant technological optimization and validation under industrially relevant conditions are still required before widespread adoption becomes feasible.

### 4.4. DNA-Based Methods for Understanding Proteins Encoded in DNA

#### Polymerase Chain Reaction (PCR) Method

Polymerase chain reaction (PCR) techniques can be utilized to detect allergenic DNA in food samples [360,361,362]. The method focuses on DNA sequences that code for allergenic proteins and is widely used in food authenticity testing [360,361,362,363,364,365]. However, PCR is less effective for allergens containing only trace amounts of DNA, such as egg and milk proteins, which may lead to false-negative results [364,365,366,367,368]. Mafra et al. [363] reviewed PCR-based approaches in food authentication, including the detection of allergens and GMOs. Their analysis showed that PCR is a valuable tool in food authentication, especially for identifying allergens and genetically modified organisms. Rodríguez- Lázaro et al. [367] emphasized the importance of real-time PCR (qPCR) as a crucial tool in food quality control and discussed strategies for utilizing this method with both nonspecific and specific detection. The authors noted that nonspecific SYBR Green fluorescent dye detection relies on binding to any double-stranded DNA. In contrast, specific detection using fluorescent oligonucleotide probes, such as TaqMan, identifies only the target DNA sequence. Bourdat et al. [369] successfully employed qPCR to detect allergens such as gluten, sesame, hazelnut, and soy, with results confirmed by ELISA.

Although PCR is a highly sensitive and specific molecular technique, it has inherent limitations that restrict its use in allergen detection. Most importantly, PCR can only identify DNA sequences encoding allergenic proteins; it does not provide information about the proteins’ presence, concentration, or structural integrity [368,369,370,371,372]. As a result, detecting allergen-encoding genes does not necessarily confirm the presence of immunoreactive proteins capable of triggering an allergic response. This limitation is especially relevant for processed foods, where proteins may remain even after DNA has degraded, or where DNA stays intact while proteins are denatured or hydrolysed. Another significant challenge is that DNA can undergo fragmentation and chemical modification during food processing. High temperatures can break phosphodiester bonds within the DNA backbone, while acid or alkaline hydrolysis and oxidation may cleave N-glycosidic bonds or damage nitrogenous bases. These structural changes weaken DNA integrity, resulting in decreased PCR sensitivity, incomplete amplification, or false-negative results [370]. Therefore, PCR results should be interpreted in conjunction with complementary analytical methods, such as ELISA or LC-MS/MS, which can confirm the presence and immunoreactivity of proteins.

In summary, various techniques for identifying allergenic proteins are illustrated in Table 4.

## 5. Technological and Economic Aspects of Reducing Protein Allergenicity

Advanced analytical and processing techniques enable accurate monitoring of physicochemical changes during protein modification and their effects on immunological properties [34]. The primary physicochemical processes that reduce protein immunoreactivity include thermal denaturation, the Maillard reaction, oxidation, and enzymatic hydrolysis [35,36,37]. When discussing the issue of protein immunoreactivity in food, it is also important to consider the economic aspect. The costs associated with allergens in food products include direct financial losses for manufacturers and serious health risks for consumers. From an economic perspective, food allergenicity incurs expenses at various levels. Production processes must be carefully managed to prevent cross-contamination, requiring secure production lines and strict allergen controls. Failure to meet these standards can lead to costly product recalls, damage to brand reputation, and the re-certification of food safety systems. For consumers, allergic reactions can result in significant medical costs, including treatment and hospitalization, and pose risks to health and life in severe cases. Therefore, effective techniques for reducing protein immunoreactivity and reliable methods for detecting and measuring immunoreactive proteins are very important. While physical food processing techniques are well-established technologically, their efficiency can sometimes be limited and may not justify their economic cost. However, chemical methods involve additional expenses linked to raw materials, reaction management, and safety validation. Furthermore, implementing advanced detection systems demands substantial investment in analytical equipment, calibration, and staff training. Moving forward, developing cost-effective, scalable technologies that uphold high safety standards while reducing production costs will be essential.

## 6. Conclusions

The increasing prevalence of food allergies, especially in children, underscores the urgent need for effective prevention, diagnosis, and management strategies. Both thermal and non-thermal food processing methods can reduce allergenicity in processed foods by modifying protein structures and immunoreactive properties, although their mechanisms require further investigation. Advanced analytical techniques, including ELISA, Western blot, LC-MS/MS, NMR, PCR, and biosensors, are essential for accurately identifying and characterizing allergenic proteins in complex food matrices. Future research should focus on developing innovative detection technologies and effective methods to lower allergenicity in food products, while validating these findings through clinically relevant studies. These efforts are crucial for enhancing food safety and protecting public health.

## Figures and Tables

**Table 1:** Doses of food products that cause hypersensitivity to 14 allergenic foods. Population EDs were estimated, where 1% or 5% of the respective allergic population was predicted to experience an allergic reaction [57,58].

Food Product	Discretea ED01 * (95% CI)	Discretea ED05 ** (95% CI)	Number of Patients
Walnut	0.03 (0.01, 0.5)	0.08 (0.10, 8.9)	74
Cashew	0.05 (0.02, 0.3)	0.80 (0.20, 5.0)	245
Mustard	0.07 (0.009, 1.1)	0.40 (0.10, 3.6)	33
Celery	0.07 (0.02, 1.9)	1.50 (0.30, 11.8)	82
Sesame	0.10 (0.03, 2.7)	0.20 (0.04, 4.8)	40
Hazelnut	0.10 (0.07, 0.6)	3.50 (1.30, 12.1)	411
Peanut	0.20 (0.10, 0.4)	2.10 (1.20, 4.6)	1306
Egg	0.20 (0.10, 0.5)	2.30 (1.20, 4.7)	431
Milk	0.20 (0.10, 0.5)	2.40 (1.30, 5.0)	450
Soy	0.50 (0.20, 3.5)	10.00 (2.20, 54.6)	87
Wheat	0.70 (0.30, 2.5)	6.10 (2.60, 15.6)	99
Fish	2.60 (1.00, 12.0)	12.10 (4.50, 43.9)	82
Lupine	2.90 (1.30, 9.1)	15.30 (6.70, 47.0)	25
Shrimp	26.20 (2.70, 166)	280.00 (69.30, 880.0)	75

* ED01—dose that will cause a reaction in 1% of the population allergic to a given allergen. ED05 **—dose that will cause a reaction in 5% of the allergic population. 95% CI—confidence interval showing the uncertainty of the estimate.

**Table 2:** Maximum allergenic protein concentrations threshold according to the FAO and WHO [60,61,62].

Food Product	Maximum Allergenic Protein Concentration Thresholds [mg/kg]
Walnuts	1
Cashews nuts	1
Almonds	1
Peanuts	2
Egg	2
Hazelnut	3
Wheat	5
Fish	5
Gluten	20
Lactose	100
Shrimp	200

**Table 3:** Effects on protein properties, advantages, and disadvantages of various food processing technologies for reducing the immunoreactivity of allergen proteins in the final food product.

Processing Technology		Effects on Protein Properties	Advantages	Disadvantages	Reference
Thermal treatment	Pasteurization Baking Cooking	Destroys hydrogen and disulfide bonds and hydrophobic interaction forces of allergens, causing allergen conformation and allergenicity changes.	- inexpensive, - popular, - convenient, - confirmed elimination of immunoreactive proteins.	- loss of nutrients - insufficient reduction in immunoreactive allergens. - amino acid oxidation and lysine degradation. - formation of neoantigens	[238,239]
Acid treatment		Denaturation of protein, change in tertiary and quaternary structures.	- increases product shelf life. - the acidic environment causes the breakdown of protein complexes and may facilitate protein isolation.	- loss of nutrients - high acidity promotes deamidation and depurination, which hinders subsequent molecular analyses.	[240,241]
Microwave Processing		Increases the temperature of the food substrate, changing the protein’s conformation and the allergen’s allergenicity.	- efficient, - fast, - preservation of nutrients.	- specialized equipment. - may accelerate Maillard reactions	[242,243]
Fermentation		Breaks down allergens into peptide and amino acid fragments, degradation of antigenic epitopes, and spatial conformation of allergens	- improvement of nutritional properties, - reduction in antinutritional substances, - may improve the sensory properties of products. - low cost	- reducing the surface charge of proteins, which promotes their aggregation and precipitation, - formation of biogenic amines, - product-specific process.	[244,245]
Nonthermal processing	High-pressure processing	High pressure can change the tertiary and quaternary spatial structure of proteins, but minimally affects the secondary structure	- change in texture - minimal impact on vitamins and nutrients, - improved protein digestibility - reduced allergenicity	- specialized equipment, - expensive method. - limited effectiveness in the case of small, stable proteins with extensive disulfide bonds	[167,246,247]
	Ultrasound	The cavitation process locally changes pressure and temperature, which can lead to protein denaturation and exposure of hidden amino acid groups of the protein	- change in consistency, - minimal impact on vitamins and nutrients, - uncomplicated method.	- high costs, - limitations: not all proteins react with ultrasound - difficulties in industrial scaling	[248,249]
	UV radiation	Change in protein structure. Amino acid oxidation may occur. A change in the expression of genes responsible for the immune response may occur	- can destroy protein structure, - fast	- prolonged exposure is harmful to health - limitations of the ability to penetrate the protein structure	[250,251,252,253]
Enzymatic hydrolysis		Provides a specific breakdown of allergenic proteins, reducing their immunoreactivity. Breakdown of proteins into amino acids or peptides	- effective reduction in protein immunoreactivity - can improve the sensory properties of products - the process is safe, - non-toxic	- requiring specific enzymes, - costly, - risk of incomplete hydrolysis, leaving immunoreactive proteins. - can reveal hidden allergenic epitopes	[254,255,256]
Cold plasma		ROS oxygen and RNS nitrogen damage proteins. Change in structure. Oxidation of amino acids	- can cause reductions in epitopes of allergenic proteins - low temperature performance - can be used in food processing - the process is safe,	- high equipment and maintenance costs, - specialized equipment, - specialized knowledge of trained individuals, - limited efficiency.	[257,258,259]

**Table 4:** Methods for detecting proteins with allergenic potential.

Technique		General Point of the Method	Advantages	Disadvantages	Reference
Test ELISA	Direct ELISA Indirect ELISA Sandwich ELISA Competitive ELISA	Detection and quantification of proteins with antibodies.	- high sensitivity and specificity, - ability to analyze multiple samples simultaneously.	- costly reagents, - requires precise execution. - false negatives due to processing-induced epitope destruction - susceptibility to matrix effects	[373,374]
PCR		A DNA-based method that is helpful in obtaining information about the protein encoded in DNA. Amplification of a specific DNA fragment to obtain a sufficient amount for further analysis using molecular methods.	-high sensitivity, -ability to analyze small amounts of genetic material.	- requires specialized equipment, - there is a risk of contamination, - costly reagents. - the need to know the sequence of the target gene - DNA isolation must be precise. - DNA degradation may occur	[362,363,364,365,375]
LC-MS/MS		Identification and quantification of proteins by liquid chromatography-mass spectrometry.	- high precision and accuracy, - ability to analyze complex mixtures of proteins. - no need to use antibodies	- high cost of equipment and reagents, - requires specialized knowledge, - time-consuming preparation. - difficulty in selecting peptide markers - lack of full standardization and reference materials	[376,377]
NMR		Analysis of protein structure by magnetic resonance.	- ability to analyze protein structure in solution, - without the need for crystallization. - ability to analyze entire mixtures without separation - high repeatability and accuracy of measurements	- high equipment costs, - requires large amounts of sample. - complex interpretation of spectra - fats, sugars, and salts may cause false results.	[378,379]
Biosensors		Protein detection with specific receptors.	- fast and direct detection, - ability to be miniaturized and automated - high sensitivity and selectivity, - requires a small sample volume and often does not require pre-treatment	- limited sensitivity and specificity, - fats, sugars, and salts may cause false results. - cross-reactivity	[352,353,354,380]
Western Blot	Standard Fluorescent Colorimetric Radioisotopic Dot Blot Far-Western Blot	Detection of specific proteins by antibodies after electrophoresis.	- high specificity, - ability to analyze post-translational modifications of proteins.	- time-consuming, - requires precise execution, - high risk of technical errors.	[381,382,383]
	Electrophoresis	Separation of proteins based on their size and electrical charge. - Not intended for self-testing for allergens.	- precise separation of proteins, - ability to analyze multiple samples simultaneously. - small sample size and quick results - easy to combine with other methods	- requires specialized equipment, - time-consuming. - no information on allergenic epitopes	[293,294,295,296,384]

## Data Availability

Not applicable.

## Abbreviations

The following abbreviations are used in this manuscript:

2D-E	Two-Dimensional Gel Electrophoresis
AF	Allergenicity Factor
ELISA	Enzyme-Linked Immunosorbent Assay
EU	European Union
FA	Food Allergy
FAO	Food and Agriculture Organization
FTIR	Fourier Transform Infrared Spectroscopy
HPP	High-Pressure Processing
IgE	Immunoglobulin E
Ka	Constant Association Rate
LAB	Lactic Acid Bacteria
LC-MS/MS	Liquid Chromatography Coupled with Tandem Mass Spectrometry
NHIS	National Health Interview Survey
NMR	Nuclear Magnetic Resonance
PCR	Polymerase Chain Reaction
PEF	Ultrasound, Pulsed Electric Field
RAST	Radio Allergo Sorbent Test
SDS-PAGE	Sodium Dodecyl Sulfate Polyacrylamide Gel Electrophoresis
TRL	Technology Readiness Level
UV	Ultraviolet
WB	Western Blot
WHO	World Health Organization
